# Stock Index Spot–Futures Arbitrage Prediction Using Machine Learning Models

**DOI:** 10.3390/e24101462

**Published:** 2022-10-13

**Authors:** Yankai Sheng, Ding Ma

**Affiliations:** School of Economics, Wuhan University of Technology, Wuhan 430070, China

**Keywords:** spot–futures arbitrage, Least Absolute Shrinkage and Selection Operator (LASSO), Extreme Gradient Boosting (XGBoost), Back Propagation Neural Network (BPNN), Long Short-Term Memory neural network (LSTM)

## Abstract

With the development of quantitative finance, machine learning methods used in the financial fields have been given significant attention among researchers, investors, and traders. However, in the field of stock index spot–futures arbitrage, relevant work is still rare. Furthermore, existing work is mostly retrospective, rather than anticipatory of arbitrage opportunities. To close the gap, this study uses machine learning approaches based on historical high-frequency data to forecast spot–futures arbitrage opportunities for the China Security Index (CSI) 300. Firstly, the possibility of spot–futures arbitrage opportunities is identified through econometric models. Then, Exchange-Traded-Fund (ETF)-based portfolios are built to fit the movements of CSI 300 with the least tracking errors. A strategy consisting of non-arbitrage intervals and unwinding timing indicators is derived and proven profitable in a back-test. In forecasting, four machine learning methods are adopted to predict the indicator we acquired, namely Least Absolute Shrinkage and Selection Operator (LASSO), Extreme Gradient Boosting (XGBoost), Back Propagation Neural Network (BPNN), and Long Short-Term Memory neural network (LSTM). The performance of each algorithm is compared from two perspectives. One is an error perspective based on the Root-Mean-Squared Error (RMSE), Mean Absolute Percentage Error (MAPE), and goodness of fit (R^2^). Another is a return perspective based on the trade yield and the number of arbitrage opportunities captured. Finally, a performance heterogeneity analysis is conducted based on the separation of bull and bear markets. The results show that LSTM outperforms all other algorithms over the entire time period, with an RMSE of 0.00813, MAPE of 0.70 percent, R^2^ of 92.09 percent, and an arbitrage return of 58.18 percent. Meanwhile, in certain market conditions, namely both the bull market and bear market separately with a shorter period, LASSO can outperform.

## 1. Introduction

The pricing model and non-arbitrage interval model of stock index futures, which were researched extensively in the 20th Century, are the foundation of the research on stock index spot–futures arbitrage. Based on the perfect market hypothesis, Cornell and French [1] presented the carrying cost model, which held that the price of stock index futures was equal to the spot price plus the discounted value of dividends. To further examine the impact of risk-free fluctuations and other variables on the prices of stock index futures, Klemkosky and Lee [2] presented the range pricing theory. From the empirical perspective, however, such evidence is not quite common. Zhong et al. [3] found that the futures market in Mexico serves the price discovery function effectively while triggering futures trading volatility in the spot market. Using intraday data for financial instruments related to the CAC 40 index, Deville et al. [4] did not identify spot–futures price efficiency improvements after Exchange-Traded Fund (ETF) introduction. Related research has become scarce recently, which may be caused by the relatively high pricing efficiency of index futures in futures markets of developed countries. In China, however, regulations have been proposed to restrict the frequent trading of index futures during the rapid fall of the A-share market in 2015, which also limited the pricing efficiency of the index futures market [5]. Hence, arbitrage opportunities may still exist.

With the development of FinTech and quantitative finance, machine learning models have been adopted extensively in the financial fields. Due to the high noise and complexity in price forecasting, time series prediction has always been a popular field of research. The application of traditional machine learning models was prevalent initially. Yu et al. proposed an Evolving Least-Squares SVM based on Support Vector Machine (SVM), which performs multiple genetic algorithm feature extraction and parameter optimization and achieves higher prediction accuracy than Auto Regressive Integrated Moving Average (ARIMA) and traditional SVM [6]. Lee et al. combined Cumulative Sum (CUSUM), Support Vector Regression (SVR), and Generalized Auto Regressive Conditional Heteroskedasticity (GARCH) to predict stock index and stock price, which presents a more promising performance than the single GARCH model [7]. Shi et al. adopted an Empirical Mode Decomposition and Grey Relational Analysis (EMD-GRA) hybrid model to construct an index representing the systematic risk in China’s stock market, which can quantify the cycle of operation of China’s stock market [8]. As deep learning models have evolved, the financial industry has also started to investigate the viability of deep learning models for time series forecasting. Fischer and Krauss were the first to use the Long Short-term Memory neural network (LSTM) in the financial fields, and they demonstrated its effectiveness in predicting financial time sequences [9]. Börjesson and Singull adopted causal and dilated convolutional neural networks to forecast the S&P500 index and acquired a low prediction error [10]. Wu et al. introduced a labeling method to determine the prediction accuracy and financial investment return, where different machine learning models, including Random Forest (RF), K-Nearest-Neighbor (KNN), LSTM, and Gated Recurrent Unit (GRU), were compared based on their labeling method [11]. Recently, machine learning models have been used in financial derivative research and have exhibited good performance. He and Wen applied a novel machine learning model to predict the riskless state of commodity futures arbitrages [12]. Ivascu compared the performance of machine learning models and parametric models in option price prediction [13]. Carta et al. constructed a trading strategy through a reinforcement learning model based on S&P 500 index futures and found it outperformed the benchmark models [14].

However, most of the studies are evidence from developed markets, while the research in developing countries is limited. Even less attention is paid to stock index spot–futures arbitrage in underdeveloped markets. China’s stock index futures market is still in an inefficient state, which leaves room for arbitrage opportunities and ensures high practical significance pertaining to the work on stock index spot–futures arbitrage prediction. Moreover, few studies have employed machine learning models in spot–futures arbitrage. Existing work is mostly retrospective, rather than anticipatory of arbitrage opportunities. In other words, most of the existing works focus on the precision of model prediction, rather than the profitability of the chosen model in practice. The over-fitting issue of machine learning models could make them less likely to generate high returns. Therefore, the comparison of machine learning models for spot–futures arbitrage performance under different investment scenarios is necessary.

To close the above-mentioned gaps, we used machine learning approaches based on historical high-frequency data to forecast spot–futures arbitrage opportunities for the China Security Index (CSI) 300. Three steps were taken before the application of machine learning models. Firstly, econometric models were adopted to test the pricing efficiency of CSI 300 futures. Secondly, an ETF-based portfolio was constructed to make the index tradable. Thirdly, a strategy based on an index we proposed was proven profitable. After that, we adopted four different kinds of machine learning models to predict the index and compared their performance, not only on the overall time sequence, but also on the bull and bear markets separately. Our contributions are threefold. First, a new index is presented that takes into account the relative position of the index futures between the upper and lower bounds of the non-arbitrage interval and serves as the foundation of the arbitrage strategy. Second, we compared the performance of various machine learning models, not only on the fitting error, but also on the returns they can forecast. Thirdly, as far as we know, we present an attempt to combine spot–futures arbitrage and machine learning methods, and our strategy was proven profitable.

This article proceeds as follows. Section 2 briefly introduces four different machine learning methods. Section 3 provides the price discovery ability of the CSI 300 futures based on the Johansen cointegration test and the Granger causality test. Section 4 introduces the construction of non-arbitrage intervals and the strategy of spot–futures arbitrage. Section 5 compares the performance of the machine learning models we adopted to predict spot–futures arbitrage opportunities. Section 6 concludes the entire work we conducted.

## 2. Literature Review

Spot–futures arbitrage is based on the prerequisite lead–lag correlation between futures and spot prices, which implies the feasibility of the arbitrage. Moreover, the construction of the spot portfolio to track the trend of futures and the derivation of arbitrage intervals from futures pricing are the key steps in the arbitrage strategy. Furthermore, machine learning has offered a promising means for the prediction of arbitrage opportunities. Correspondingly, the literature review is sorted into four parts, including the spot–futures relationship, spot portfolio construction, futures pricing and arbitrage intervals, and arbitrage prediction using machine learning models.

### 2.1. Spot–Futures Relationship

Through investigating the lead–lag relationship between the futures and the spot, the pricing information efficiency of the market can be inferred. Generally speaking, there is a strong linkage between the futures price and the spot price. If the deviation between the two is too large, it will trigger arbitrage transactions and promote the return of equilibrium. Econometric models are commonly used methods to analyze the relationship between the two. Kawaller et al. [15] used minute-level high-frequency data to test the relationship between intraday price changes of the S&P500 index spot and futures, and the results proved that there is a significant synergistic relationship between the two. They also found that futures price changes always lead the index changes by 20 to 45 min, while movements in the index rarely affect futures for more than 1 min. Chan [16] found that asynchronous trading could not fully explain the lead–lag relationship between futures prices and spot indices. Abhyankar [17] identified that the reason why the futures price is ahead of the spot price is that the futures transaction is relatively low-cost and high-liquidity and has a fast transaction speed. In addition, based on the factor of lower transaction cost, Booth et al. [18] found that the leverage ratio is also an important factor to produce the lead–lag relationship. After China launched the CSI 300 futures, relevant studies based on this research object have not reached a consensus due to different sampling periods or methods. Zhang et al. [19] distinguished the trend of price changes and identified that stock index futures have the function of price discovery in an uptrend, while in a downtrend, stock index futures and the spot have mutual Granger causality. Huang et al. [20] found that the futures market is in a dominant position in terms of price discovery ability in both the rising stage and the falling stage. Xu and Liu [5] analyzed the impact of trading restrictions on the spot–futures relationship and found that, before the implementation of trading restrictions, stock index futures had a stronger impact on stock market prices, especially during periods of sharp price declines. They explained that the trading policy has significantly increased the transaction cost of the futures market, thereby reducing the information share of the futures market, weakening its price impact on the stock market and, consequently, changing the impact model of the futures price on spot price.

### 2.2. Spot Portfolio Construction

To carry out the spot arbitrage of stock index futures, it is necessary to have the underlying index spot corresponding to the stock index futures contract. Since the underlying index is not tradable, the construction of the corresponding spot portfolio is indispensable. Existing research mainly obtains higher returns through spot portfolios with higher fitting accuracy, minimum tracking error, convenient transaction, and lower cost. There are two commonly used methods for constructing spot portfolios in the existing literature. One is the replication method of constituent stocks, and the other is the construction method of the ETF. Andrews et al. [21] proposed three replication combination methods for arbitrage on index futures, namely full replication, sampling replication, and hierarchical replication. Meade and Salkin [22] proposed that the method of quadratic programming in minimizing tracking error to obtain the portfolio weights has the best tracking effect and that too many constraints will weaken the tracking effect. Aiming to address the poor long-term tracking effect of the previous method, Carol and Anca [23] used the cointegration method to minimize the price difference between the target index and the tracking portfolio. Jansen and van Dijk [24] constrained the number of stocks in the tracking portfolio and used the continuous tracking error as the weighted objective function and the continuous function to approximate the discrete part and, finally, employed the standardized quadratic programming method to optimize the weight of the stocks in the selected tracking portfolio. Using the underlying index of HuaAn Shangzheng 180ETF and E-Fund Shenzheng 100ETF as the spot portfolio, Zhang and Fang [25] identified that IF1005 and IF1102 were the two main contracts having unilateral arbitrage opportunities.

### 2.3. Futures Pricing and Arbitrage Intervals

In reality, there are transaction costs, impact costs, and tracking errors in stock index spot–futures arbitrage, so there exists a non-arbitrage interval. Only when the deviation between stock index futures and spot stock is outside the non-arbitrage interval can arbitrage be profitable. The analysis arbitrage interval is commonly based on the carrying cost pricing model. Cornell and French [1] proposed a carrying cost pricing model based on the efficient market hypothesis. The authors further introduced factors such as dividends and taxes to give an extended form of the model. Modest and Sundaresan [26] added factors such as transaction costs into the carrying cost pricing model and derived non-arbitrage intervals. Klemkosky and Lee [2] further considered factors such as the interest rate of borrowed funds, transaction costs, and dividend payments and calculated the upper and lower boundaries of the non-arbitrage intervals through the combination of bid and ask spreads. This model established the foundation for the spot–futures arbitrage studies. Some scholars employ general equilibrium models. Hemler and Longstaff [27] developed a closed-end equilibrium pricing model for stock index futures through adding the stochastic form of interest rates and market volatility into the pricing model and empirically found that market volatility has a significant impact on stock index futures prices. For CSI 300 spot–futures arbitrage, Li and Chen [28] used high-frequency data analysis and found that, since the listing of CSI 300 futures, there have been decreasing arbitrage opportunities, the single arbitrage income has dropped rapidly, and the duration of arbitrage opportunities has become shorter. Based on the model of Klemkosky and Lee [2], Liu and He [29] took into account factors such as transaction costs, impact costs, margin financing, and securities lending and deduced a stock index futures pricing model that conforms to the domestic market conditions. Xie and Li [30] used the principle of no-arbitrage to conduct an empirical analysis on the price law of the CSI 300, SSE 50, and CSI 500 futures and found that the prices of the three major stock index futures are relatively low, but the prices will tend to be reasonable when the expiration date is approaching. Among them, the price determination mechanism of the Shanghai Stock Exchange 50 futures is relatively mature.

### 2.4. Arbitrage Prediction Using Machine Learning Models

The existing literature on arbitrage often carries out statistical arbitrage based on historical data. The arbitrage strategy is to trade on the spread between assets, so the prediction of the spread is critical. The prior research is mostly based on the mean reversion principle of the spread, in which the cointegration is often used to determine the feasibility of arbitrage [31]. Since the spread series is often nonlinear, traditional financial time series methods often fail to predict it, and machine learning has unparalleled advantages in dealing with nonlinear data. Therefore, machine learning is increasingly used in constructing arbitrage strategies, and neural networks comprise the mainstream method. The research employing neural networks in futures arbitrage mostly uses the Back Propagation (BP) neural network [32,33]. The BP neural network is a feedforward-type model, which has certain advantages in the prediction of nonlinear price data, but it also has shortcomings such as a slow convergence speed and insufficient prediction accuracy. On this basis, scholars began to study the application of more efficient recurrent neural network models in this field such as the Long Short-Term Memory (LSTM) neural network model proposed in 1997 [34]. Long et al. [35] used LSTM to predict the spread of coke futures, iron ore futures, and rebar futures and established an arbitrage strategy, and the results confirmed that the LSTM neural network is superior to the BP neural network and the convolutional neural network. Besides, some ensemble learning models are also used in arbitrary prediction. Zhou [36] used a rolling sample window to predict the intertemporal spread of commodity futures by three machine learning methods including the neural network, support vector regression, and XGBoost. The result indicated that the support-vector-regression-based arbitrage model can achieve significantly better performance in terms of returns and the winning rate. The gaps of the relevant studies and the potential contributions are summarized in Table 1.

Based on the current literature, we firstly tested the pricing efficiency of the CSI 300 futures through a cointegration test and Granger causality test, in which the results were indicative of low pricing efficiency and the existence of arbitrage opportunities. Then, based on the smallest tracking error principle, the ETF portfolio was built to fit the trend of the spot index so that a tradable spot index was constructed. A non-arbitrage interval was adopted, and a new index serving the function of a strategy indicator is proposed as a result. A strategy was constructed and proven profitable based on parameter optimization. Four different machine learning models were used in forecasting the strategy index, based on which both the fitting errors and performance were compared.

## 3. Pricing Efficiency of CSI 300 Futures

In this section, we hope to reveal the pricing efficiency of the CSI 300 futures. We firstly acquired the raw data of the China Security Index (CSI) 300 and its futures price, where a stationarity test is necessary. On this basis, we have a cointegration test to observe the price efficiency of the index futures. Then, the Granger causality test shed light on the leading–lagging relationship between the futures and spot prices of CSI 300. Finally, quantile regression helps us distinguish the relationship under different market circumstances.

### 3.1. Data Acquisition and Stationarity Test

**Figure 1:**
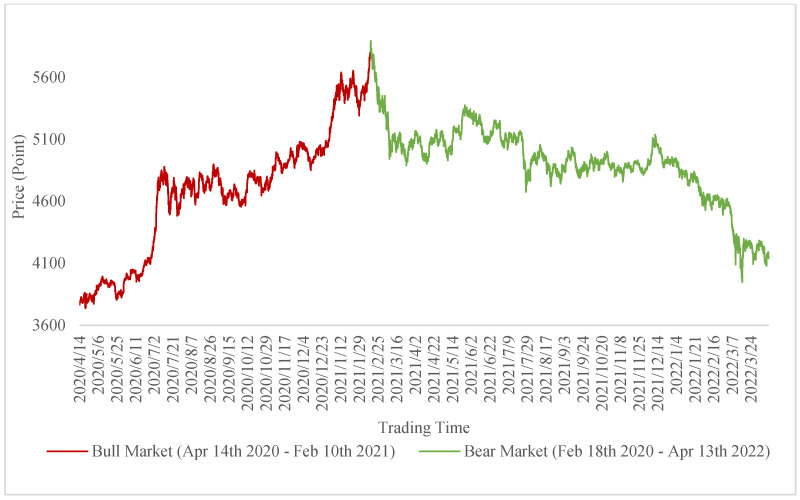
The trend of CSI 300 and separation of the market.

As the first equity index launched by both the Shanghai Stock Exchange and Shenzhen Stock Exchange, the CSI 300 has become an effective tool to reflect the price fluctuation and performance of China’s A-share market [37]. Hence, we adopted CSI 300 (Code: 000300.XSHG) and CSI 300 Futures (Code: IF9999.CCFX) as the research objects. Due to the better performance of forecasting using high-frequency data [38], this research acquired 5 min market data from JQData through Python to conduct the experiments. We found that the CSI 300 from 14 April 2020 to 13 April 2022 can be separated into two stages, as shown in Figure 1. Since our study included comparing different machine learning models on separate periods, this dataset is appropriate to serve such a research purpose.

The input of the cointegration test must be an unstable time sequence [39], while the requirement of the Granger causality test [40] is the opposite. Therefore, before the cointegration test and Granger causality test, a stationarity test is necessary, and the ADF test is the most widely used. To eliminate the heteroscedasticity, the logarithm of the raw sequence is necessary. The result of the Augmented Dickey–Fuller (ADF) test is shown in Table 2.

In Table 2*, lns* and *lnf* are defined as the logarithm to the raw sequence of spot index and futures price, while *d_ln*s and *d_lnf* are defined as the logarithmic difference of the raw sequence of spot index and futures price. The ADF test, namely the unit root test, tests whether there is an Auto Regression (AR) process with a lag term coefficient of 1. When the unit root exists, the relationship between the independent variable and the dependent variable is deceptive, because any error in the residual series will not decay with the increase of the sample size. Thus, the effect of the residual in the model is permanent. This kind of regression is also called pseudo-regression. If the unit root exists, the process is a random walk. If the sequence is stationary, there is no unit root; otherwise, there will be a unit root. Therefore, the null hypothesis of the ADF test is the existence of a unit root. If the significance test statistic obtained is less than three confidence levels (10%, 5%, 1%), the null hypothesis should be rejected with certainty (90%, 95, 99%). The first row is the ADF test statistics of all the sequences. The second row is the *p*-value of parameter estimation, where greater than 0.1 or 10% indicates that the null hypothesis could not be rejected. The last three rows are the critical values of the test statistics at the three significance levels, that is 1%, 5%, and 10%. If the t test statistics are less than the critical values, the probability of the occurrence of the null hypothesis is less than the corresponding significance levels. For example, the ADF statistic *lnf* is −2.190, which is larger than the 10% level (−2.567), and the *p*-value is larger than 0.1 or 10%. Thus, it is unstable. The conclusion can be drawn that the sequences of *lns* and *lnf* are unstable, while the sequences of *d_lns* and *d_lnf* are the opposite.

### 3.2. Cointegration Test

If CSI 300 futures and CSI 300 spot prices are highly correlated and exhibit a lead–lag relationship, CSI 300 futures have a pricing efficiency in that they lead to the change of the spot price. Intuitively, a relatively weak correlation implies the existence of arbitrage opportunities. The Johansen cointegration test [39] is used to test the correlation between CSI 300 futures and CSI 300 spot prices. Since cointegration is conducted on unstable time series, we chose the sequences of *lns* and *lnf* to perform the test. A prerequisite step of the Johansen cointegration test is to determine the optimal lag order, as it is very sensitive to the lag period. Generally, the Akaike Information Criterion (AIC) and Bayesian Information Criterion (BIC) of the Vector Auto Regression (VAR) model, based on the theory of Maximum Likelihood Estimation (MLE), are used in lag determination, in order to make the model more accurate and less complicated. The smaller the values of the AIC and BIC, the better the cointegration models are. In Table 3, from the comparisons of the AIC and BIC statistics under different lags of VAR, the lags of 4 and 7 correspond to the smallest AIC and BIC values.

We conducted the Johansen cointegration test twice under both lag settings. The results of the experiments are shown in Table 4. Both the trace test and maximum eigenvalue test demonstrated that one cointegrating vector is suitable. However, when lag = 7, the residual of the Vector Error Correction Model (VECM) was not autocorrelated, while lag = 4 did not have this character. Thus, the model with lag = 7 is more effective. When the coefficient of cointegration approaches the upper and lower bounds of (1, −1), it means that the two time series are highly related. In our study, the coefficient of cointegration was (1, −0.98806), which deviated further from (1, −1) compared with those in the markets of America and Europe. In other words, the pricing ability of CSI 300 index future is relatively weak, which indicates arbitrage opportunities.

### 3.3. Granger Causality Test and Quantile Regression

The correlation coefficient between the CSI 300 spot and futures prices reached 0.999. However, the leading relationship between these two indexes should be distinguished so that arbitrage opportunities may exist theoretically. We can identify this relationship using Granger’s [40] causality test, which has been shown to be effective in identifying the relationships between the finance and insurance sectors [41] and illustrates the relationship between stock return and implied volatility [42]. We conducted the Granger causality test using the sequences of *d_lns* and *d_lnf* due to their stability. The result is shown in Table 5. The chi2 value is indicative of whether the time series satisfy the hypothesis. If it is significant under the 5% level, the null hypothesis can be rejected, suggesting that the futures price is the Granger cause of the spot index. Therefore, the trend of the CSI 300 futures can lead the trend of CSI 300.

**Figure 2:**
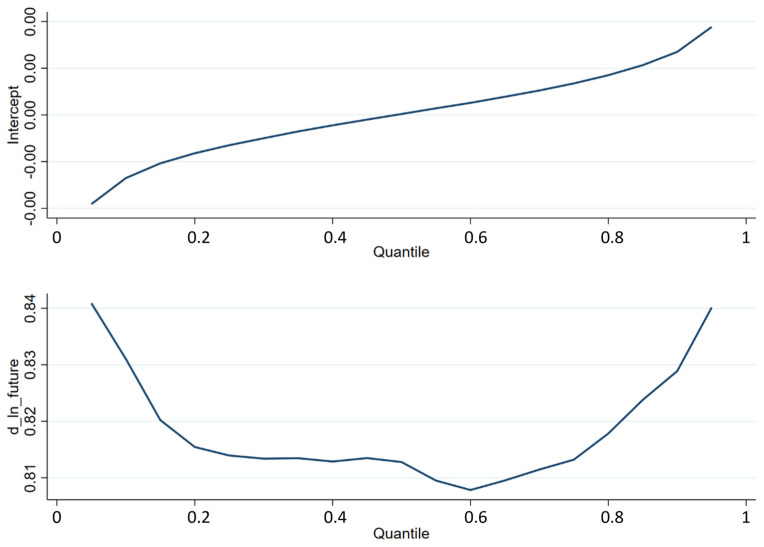
Result of quantile regression. Note: d_ln_future means the regression coefficient between the quantile of the yield of the spot index and the logarithmic difference of the index futures. The upper part of this figure reveals the trend of the intercept of quantile regression, while the lower part illustrates the change of the regression coefficient.

After the Granger causality test, quantile regression is the follow-up analysis. Quantile regression was proposed by Koenker and Basset in 1978 to address the concern pertaining to OLS in falling to illustrate the relationship between the explanatory variable and the explained variable in different intervals [43]. Our study set the highest quantile at 95%, the lowest at 5%, and the interval between each quantile at 5%. Figure 2 shows the quantile regression results, and it is clear that the leading relationship of the futures price is stronger when the market is experiencing a sharp rise or fall, where there may be an opportunity for arbitrage. This relationship was tested using historical market prices.

## 4. Strategy of Spot–Futures Arbitrage and Its Application

In this section, we first constructed a spot portfolio using Exchange-Traded Funds (ETFs) to fit the trend of the China Security Index (CSI) 300 based on the minimum Tracking Error (TE) criterion. Then, a non-arbitrage interval and appropriate parameter settings were chosen based on the existing literature and the current situation of Chinese stocks and the futures market. After that, a new index is presented, so that the best opportunity to open and unwind positions is easy to distinguish. Based on this strategy, we back-tested the arbitrage opportunity of CSI 300 and its futures from 14 April 2020 to 13 April 2022.

### 4.1. Construction of Spot Portfolio

**Figure 3:**
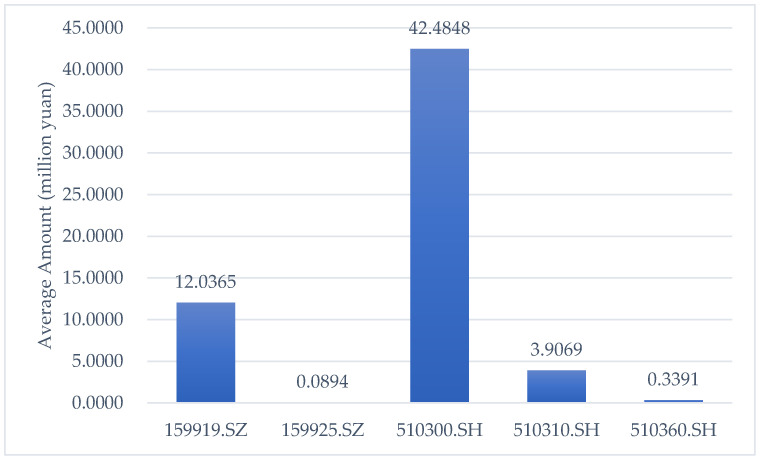
Average trading amount of each ETF. Note: The x-axis represents the names of the ETFs, and the y-axis denotes the average trading amount over the sampling period.

Since CSI 300 cannot be traded directly, a spot portfolio needs to be constructed to fit the trend of the index. CSI 300 is generated by 300 stocks based on a combination of different weights. Therefore, the direct purchase of a stock combination will incur extremely high contract fees. We chose the most widely used strategy, the ETF-based strategy, to construct the portfolio, which has been demonstrated to have a higher Sharpe ratio [44] and risk premium due to the volatility it introduces [45]. There are six main ETFs tracking the trend of CSI 300, whose codes are 159919.SZ, 159925.SZ, 510300.SH, 510310.SH, 510360.SH, and 515130.SH. The data we acquired are partly shown in Table 6. Considering the period chosen and the first trading day of each ETF, we removed 515130.SH from the portfolio, which was established on May 21st, 2020. Moreover, the liquidity of each ETF must be considered. We adopted the daily average trading amount over the sampling period to compare the liquidity of ETFs, which is shown by the bar chart in Figure 3. It is obvious that 159925.SZ and 510360.SH have low averages, so that they can be ignored in the portfolio’s construction. Therefore, these two ETFs can also be removed.

On the basis of the selection of the ETFs, the TE of the portfolio needs to be calculated so that the best portfolio can be chosen. In this research, we adopted a more commonly used method to the calculate tracking error of the portfolio in practice, whose equation is shown in (1).

(1) TE=∑t=1n[(Rt–rt^)–∑t=1n(Rt–rt^)n]2(n–1)

In this equation, *TE* is the tracking error of the portfolio; $R_{t}$ means the yield of CSI 300 on the *t*th day; $\hat{r_{t}}$ means the estimator of the yield of the portfolio on the *t*th day. *n* is the sample size (*n* equals 23,280 and represents the total rows of 5 min market data collected). We adopted Ordinary Least Squares (OLS) to calculate the weight of each ETF in the portfolio and the tracking error. The equation is shown in (2).

(2) $$R_{t}{=\alpha +\beta }_{i}{r\_\mathrm{ETF}}_{i,t}+\mathrm{TE}$$

In this equation, α is the constant term; β*_i_* is the weight of the *i*th (*i* = 1, 2, 3) ETF; *r_ETF_i,t_* is the yield of the *i*th ETF on the *t*th day. The result of OLS is shown in Table 7. The conclusion can be drawn that No. 7 is the best portfolio because the *TE* of the portfolio is the smallest and the *R*^2^ is the biggest.

Finally, we tested the correlation coefficient of the trend of the ETF portfolio constructed with the trend of CSI 300. The value we obtained was 0.995, demonstrating the high correlation of our portfolio and the index. Through duplicating a tradable index by the ETFs’ portfolios, we constructed a spot portfolio to fit the trend of CSI 300.

### 4.2. Determination of Non-Arbitrage Interval

Based on the non-arbitrage condition proposed by Modest and Sundaresan [26], the quantified cost of transactions proposed by Klemkosky and Lee [2], and the further combination of this model with China’s A-share and futures market researched by Cao [46], we adopted the non-arbitrage interval shown in Equation (3). The construction of the non-arbitrage interval was based on the following assumptions: (1) the trading cost and impact cost are invariant; (2) the short mechanism is allowed, and the rate of the security loan is also invariant; (3) the market is efficient, where the investors are completely competitive.

(3) $$\frac{S_{t}\left(1-2C_{s}-r_{c}\right){\left(1+r\right)}^{T-t}-D{\left(1+r\right)}^{T-t}-\frac{2C_{f}\left(1+2C_{fc}\right)+C_{f}(1+C_{fc}){\left(1+r\right)}^{T-t}}{z}}{1+2C_{fc}+b(1+C_{fc})[{\left(1+r_{b}\right)}^{T-t}-1]}<F_{t}<\frac{S_{t}\left(1+2C_{s}\right){\left(1+r\right)}^{T-t}-D{\left(1+r\right)}^{T-t}+\frac{2C_{f}\left(1-2C_{fc}\right)+C_{f}(1-C_{fc}){\left(1+r\right)}^{T-t}}{z}}{1-2C_{fc}-b(1-C_{fc})[{\left(1+r_{b}\right)}^{T-t}-1]}$$

The explanation of the symbols and the parameter setting are shown in Table 8. Several points need to be explained in detail. Firstly, we conducted arbitrage transactions from the perspective of institutional investors. Thus, the trade cost of the spot portfolio and futures was set at the lowest level we could find in the Chinese market, so as the rate of the securities loan. Secondly, the dividend rate of each ETF was calculated through the dividend condition of each ETF disclosed every year. Then, the dividend rate of the whole portfolio was calculated by a weighted average based on the weight of each ETF in the portfolio. Thirdly, as mentioned above, we confirmed the non-arbitrage interval from the perspective of institutional investors. Besides, the market data we acquired are high-frequency. Hence, the adoption of the overnight Shanghai Interbank Offered Rate (SHIBOR) as the rate of the borrowed funds is rational.

### 4.3. Strategy of Arbitrage and Its Back Test

It is obvious that when the price of the futures is higher than the upper bound of the non-arbitrage interval or lower than the lower bound of the non-arbitrage interval, the opportunity for arbitrage exists. However, the best time to unwind positions is difficult to decide. Therefore, we propose a new index named *RP* referring to min–max standardization, quantifying the relative position of the price of the CSI 300 futures between the upper bound and the lower bound of the non-arbitrage interval, which is calculated as Equation (4) to solve the problem mentioned above.

(4) $$\mathrm{RP}\equiv \frac{F-\mathrm{FL}}{\mathrm{FU}-\mathrm{FL}}\times 100$$

**Figure 4:**
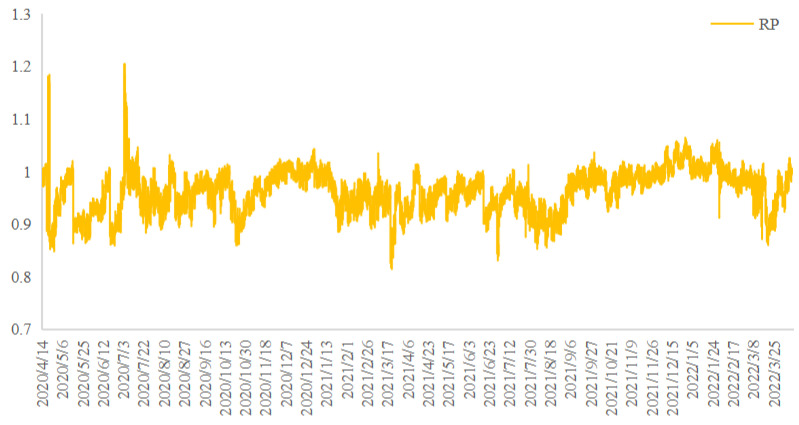
Trend of *RP*.

In this equation, *F* is the price of the CSI 300 futures. FL and FU are the lower bound and upper bound of the non-arbitrage interval, respectively. The trend of *RP* is shown in Figure 4.

It is easy to understand when *RP* > 1, *F* > *FU*, which declares the opportunity to buy the spot portfolio and sell index futures. However, how to use the *RP* index to decide when to unwind positions should be considered. The best way to find this value is to try every min (*RP*) < *RP* < 1 as the signal to unwind positions and discover under which circumstances the total return of this trade will be the highest. We conducted a search algorithm based on the target shown in (5).

(5) $$\max\sum^{\;}(\frac{S_{T}{–S}_{t}}{S_{t}}+\frac{F_{t}{–F}_{T}}{S_{t}}–C)\;$$

In this equation, *t* means the time to open positions; *T* means the time to unwind positions; C is the cost of trading, including the cost of spot portfolio trading and index futures trading. The first part $\frac{S_{T}{-S}_{t}}{S_{t}}$ is the return from the long position of the portfolio we constructed in Section 4.1. $\frac{F_{t}{-F}_{T}}{S_{t}}$ is the return from the short position of index futures. We would like to decide under which circumstance or, more clearly, what value of ${\mathrm{RP}}_{n}$ signals the opportunity to unwind positions to gain the highest return. Assume that *RP_n_* is the best *RP* value to unwind positions. The result of this search is shown in Table 9. This table declares that, when we unwind positions at *RP_n_* = 0.99886, the total return will be the highest. This index was used because we needed to acquire the return based on the predicted data of the machine learning models, while the highest return based on the primitive data had a referential value.

**Figure 5:**
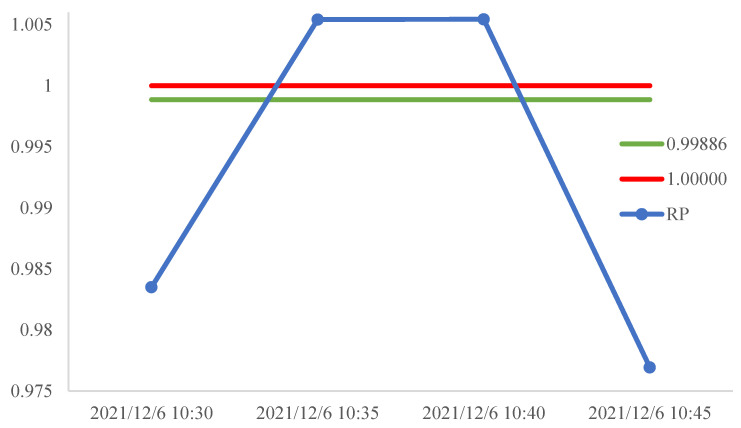
*RP* from 10:30 6 December 2021 to 10:45 6 December 2021.

To explain our strategy, an example is given below. The *RP* index in the period from 10:30 6 December 2021 to 10:45 6 December 2021 is shown in Figure 5.

## 5. Arbitrage Transaction Based on Machine Learning Model

### 5.1. Performance of Machine Learning Model

We adopted the Least Absolute Shrinkage and Selection Operator (LASSO), Extreme Gradient Boosting (XGBoost), Back Propagation Neural Network (BPNN), and Long Short-Term Memory neural network (LSTM) to forecast the arbitrage opportunity, as they are representative of linear machine learning models, ensemble learning models, neural network models, and deep learning models. When we reviewed the previous literature, we found that, in recent years, the ensemble learning and deep learning models are topical issues in relevant research, while the linear models and neural networks may not be so popular. However, when they were created, their promising performance gained great attention. Therefore, we would like to show the comparison between state-of-the-art models and classical models. More details of the algorithms are presented in the Appendix A. We chose to predict *RP*, since it is the most direct index, and we can identify the transaction opportunity. If we predict CSI 300 and the price of the CSI 300 futures instead, the non-arbitrage interval will be too hard to construct, and a larger error will be introduced. We used the data of the *RP* index of the last ten days as our input, and the output was its data for the next day. All of our research was conducted on Python 3.9.7 and the TensorFlow 2.8.0 Library. The main parameter setting of these machine learning models is shown in Table 10, which were all based on the grid search of the lowest error.

Apart from the parameter setting, the performance indicator of our study is shown in Equations (6)–(8). The Mean-Squared Error (*MSE*) is more popular to evaluate the performance of the model. This research adopted the Root-Mean-Squared Error (*RMSE*), which is not only the square root of the *MSE*, but also has the same magnitude as the raw data. The *MAPE* is another indicator to evaluate the accuracy of the model based on the absolute error between the forecasted value and the real value. Most econometric models adopt *R*^2^ (goodness of fit) as the estimator of the explanatory ability of the model. Here, we also adopted *R*^2^ to acquire the fitting effect of each model numerically.

(6) $$\mathrm{RMSE}=\sqrt{\frac{\sum_{i=1}^{n}{\left(\hat{y_{i}}{-y}_{i}\right)}^{2}}{n}}$$

(7) $$\mathrm{MAPE}=\frac{\sum_{i=1}^{n}\left|\frac{\hat{y_{i}}{-y}_{i}}{y_{i}}\right|}{n}\times 100\%$$

(8) $$R^{2}=1-\frac{\sum_{i=1}^{n}{\left(\hat{y_{i}}-y_{i}\right)}^{2}}{\sum_{i=1}^{n}{\left(y_{i}-\bar{y}\right)}^{2}}$$

In Equations (19)–(21), *n* is the size of the test set; $\hat{y_{i}}$ means the *i*th forecasting value of the machine learning model; *y*_i_ means the *i*th real value; $\bar{y}$ means the average value of the real data. We split the whole set into a training set and a test set, whose proportion was 80% and 20%, respectively. To be more specific, the test set was the last 20% of the RP index, which was the RP index from 10:40 18 November 2021 to 15:00 13 April 2022. The result of these indicators of each model is shown in Table 11. Based on the test set, the BPNN performed worst with the highest RMSE and MAPE and lowest R2. One explanation is that the processing of the BP neural network at each moment is independent, which is inconsistent with the case of time series. If it is used in the arbitrage strategy, it may miss the opportunity to build and unwind arbitrage positions. LSTM was the best forecasting model with the lowest RMSE and MAPE and highest R2, because LSTM evolved from recurrent neural networks, which is more suitable for long time sequence. XGBoost, firstly created to solve classification problems, performed worse than LSTM. LASSO, however, being the simplest form of model, had a surprisingly good performance. The reason for the better performance of LASSO in a short period may lie in the fact that LASSO evolved from OLS, which performs better on small samples.

**Figure 6:**
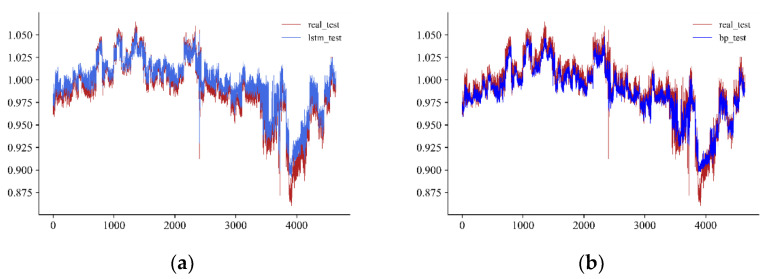

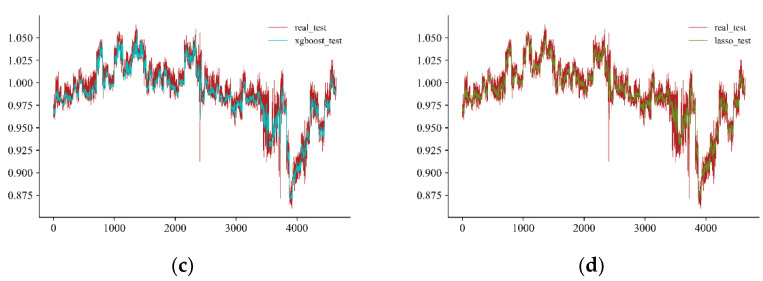
Real trend of *RP* and fitted trend of each algorithm. Note: Subfigure (**a**–**d**) represents the real trend of *RP* and the fitted trend of LSTM, BPNN, XGBoost and LASSO respectively.

In addition, we exported the comparison figures of the fitted curve and real curve based on the test set. Figure 6 illustrates the result that the fitting effect of LSTM was the best, which can reflect the most fluctuations of the real curve.

### 5.2. Arbitrage Return Using Machine Learning Model

To further compare the performance of all these machine learning models, we calculated the total arbitrage return and the number of trades based on the *RP* predicted by each algorithm in the test set. Due to the promising predicting performance, we traded at the price at the time of the signal. Apart from the assumptions of trading mentioned in Section 4.2, here, we also needed to assume that, if the model signals the arbitrage opportunity, we can open a position immediately at the price of the signal. The result is shown in Table 12. Arbitrage transactions based on the real data obtained a yield of 58.25% and conducted 307 transactions. *RP* predicted by LSTM signaled 277 trades whose return reached 58.18%, being quite close to the real situation. However, the other algorithms did not show such promising results. This result is not surprising, which matched the error data shown in Table 11. At the same time, it can be found that, although the XGBoost and LASSO models had better fitting effects and smaller errors than the BPNN, their yields in practice were not as good as the BPNN. From the trend comparison in Figure 6, it can be seen that the predictions of LSTM and the BPNN fluctuated more frequently and could send out trading signals more accurately, so they are advantageous from the perspective of profit.

**Figure 7:**
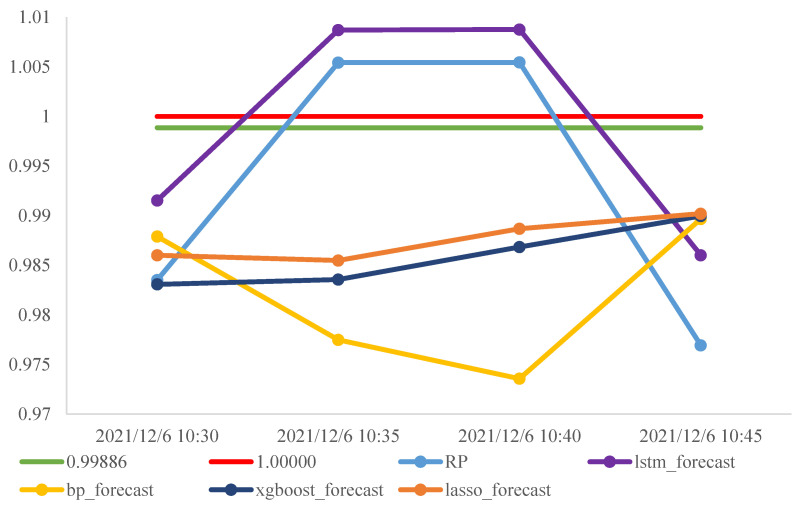
Real trend of *RP* and forecasted trend of machine learning methods in a 15 min period.

**Figure 8:**
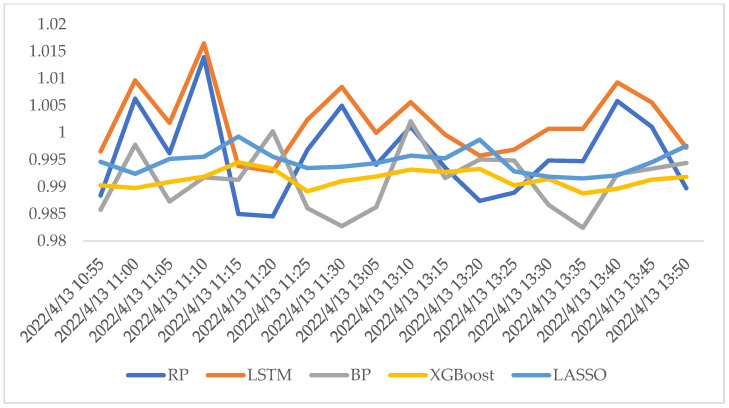
The comparison of the predicted value and true value of RP in a 95 min period.

We also followed the example we proposed in Section 4. The result is shown in Figure 7, where the predicted data of all machine learning models in the interval from 10:30 6 December 2021 to 10:45 6 December 2021 are presented. In this period, the real *RP* index signaled the time to open positions at 10:30 6 December 2021 and unwind positions at 10:45 6 December 2021. The same decision was made by the LSTM algorithm, while the other algorithms did not signal the trade opportunity. Therefore, LSTM was the best model based on this period. Apart from this short period, we also present a relatively long period. We chose the interval 10:55 13 April 2022 to 13:50 13 April 2022 as an example, whose result is shown in Figure 8. From Figure 8, the promising performance of LSTM can be proven again. The RP index dropped sharply at 11:15 and increased to 1.000 at 11:30, which was only tracked by LSTM. Furthermore, RP increased to 1.005 at 13:40, but dropped rapidly in the interval of 13:45 to 13:50, which was also only predicted by LSTM.

Next, we offer a comparative perspective of the different statuses of the stock market. This comparison was based on the separation of markets mentioned in Figure 1.

### 5.3. Comparison of Machine Learning Methods under Different Status of Stock Market

#### 5.3.1. Bull Market from 14 April 2020 to 10 February 2021

In the period of 14 April 2020 to 10 February 2021, CSI 300 increased by 51.81%, which we assumed was a bull market. In this period, however, the *RP* index witnessed a wild fluctuation. Compared with the whole period, LSTM performed relatively poorly, while LASSO showed a surprisingly good performance (Table 13). Further analysis would be conducted if the same phenomenon happened in the bear market.

#### 5.3.2. Bear Market from 18 February 2021 to 13 April 2022

In the period of 18 February 2021 to 13 April 2022, CSI 300 decreased by 28.72%. From Table 14, we find that LASSO outperformed the other machine learning models. The reason is that, being the only model evolved from linear regression, LASSO can perform better on a relatively small dataset and has a more certain tendency. LSTM, however, can extract more information based on a long period and a large dataset due to its recurrent character and its memory mechanism.

## 6. Conclusions

In this research, we used machine learning methods to predict spot arbitrage opportunities. Firstly, through the cointegration test, Granger causality test, and quantile regression, we found that the price efficiency of the CSI 300 futures is still low, demonstrating that an arbitrage opportunity may exist. Meanwhile, the strong leading relationship between futures and spot indexes during sharp rises and falls can ensure the return on each arbitrage transaction. Next, we constructed a spot portfolio of ETFs to fit the trend of CSI 300 using OLS. Based on the previous literature, a non-arbitrage interval was decided. The strategy we adopted to capture the opportunity of a transaction was to construct an index, *RP*, so that further work can proceed on this basis. Finally, a comparative perspective of our research was provided based on the error and arbitrage return of each machine learning model. The conclusion was that the LSTM neural network performed best over a long period, while LASSO was better if the dataset was relatively small.

Overall, we successfully combined the machine learning models with the spot–futures arbitrage and proved that a high return can be earned through an arbitrage strategy and deep learning model. Future work can be conducted to identify other types of arbitrages with the application of machine learning models and comparing the differences of the mechanisms and the performance of each model. There are some limitations that remain to be resolved in futures studies. We only presented the performance of different primitive machine learning models, while the exploration of an improved model or the proposal of a new model is lacking. Moreover, with the improved model, we may conduct further out-of-sample predictions to make this research more practical.

## Figures and Tables

**Table 1:** Summary of relevant studies.

Relevant Studies	Gap and Potential Contribution
Unit root tests and autoregressive multivariate cointegration models were used to test the relationship among hog, corn, and soybean meal futures price series, and the cointegration results indicated considerable arbitrage profit [31].	Traditional econometric models rarely consider the compatibility between models and data, which often leads to the dual problems of complex models, but unsatisfactory prediction results. Machine learning does not emphasize the structure of the model and only needs to check the accuracy of the prediction according to the input data, so it can better adapt to the characteristics of the rapid change of financial markets and the complex data structure. Therefore, machine learning was employed in this study and proven feasible in identifying arbitrage opportunities.
The BP neural network and convolutional neural networks were used in forecasting the prices of Shanghai zinc futures [32].	The BP neural network is a back propagation neural network, and its processing at each moment is independent, which is inconsistent with the case of time series. It has the disadvantages of a slow convergence speed and low prediction accuracy. If it is used in the arbitrage strategy, it may miss the opportunity to build and close arbitrage positions. The LSTM neural network is a recurrent neural network, which can feed back the output at time t to the input at the next time, and it can better extract the information of time series. This study focused on comparing the arbitrage strategy based on BP and LSTM and found that LSTM performed better in the prediction of the RP index established in this study.
The arbitrage strategy of ferrous metal futures based on the LSTM neural network is feasible and effective and performed better than the BP neural network and the convolutional neural network [35].	This study did not carry out a comparative analysis of strategies under the state of market separation. We added the performance heterogeneity analysis of the bull market and bear market, so as to better judge the performance of the model in different market states. The applicability of different machine learning models in the field of financial investment is quite different. This paper focuses on the comparison of the LASSO, XGBoost, and neutral network models. The results also showed that the LASSO model performed well on short datasets.
By predicting the spread in the intertemporal arbitrage of commodity futures, the author proved that SVR performs better than the traditional arbitrage model. When using the standard distance method to set an arbitrage threshold, the winning rate increased, but the return decreased [36].	Most studies conduct position building and unwinding by predicting the change of the spread and setting the threshold, which involves the adjustment and change of many parameters and inevitably brings practical difficulty. The RP index in this study was set to judge the timing of opening positions, and the best liquidation RP value was determined by the traversal method. The process is simple and straightforward, and the winning rate and income were also satisfactory. It can be said that this paper provides a new idea for how to determine the timing of opening positions, especially unwinding positions.

**Table 2:** Result of ADF test of CSI 300 and CSI 300 futures.

Index	CSI 300		CSI 300 Futures	
	*lns*	*d_lns*	*lnf*	*d_lnf*
ADF statistic	−2.161	−86.872	−2.190	−87.233
p statistic	0.221	0.000	0.210	0.000
1% level	−3.431	−3.431	−3.431	−3.431
5% level	−2.862	−2.862	−2.862	−2.862
10% level	−2.567	−2.567	−2.567	−2.567

**Table 3:** AIC and SBIC statistics under different lags.

Lag	AIC	BIC	Lag	AIC	BIC
0	−10.4780	−10.4773	7	−21.7033 *	−21.6929
1	−21.6165	−21.6144	8	−21.7032	−21.6914
2	−21.6895	−21.6861	9	−21.7030	−21.6899
3	−21.6974	−21.6926	10	−21.7027	−21.6882
4	−21.7000	−21.6938 *	11	−21.7026	−21.6867
5	−21.7012	−21.6936	12	−21.7024	−21.6851
6	−21.7016	−21.6926	13	−21.7023	−21.6836

Note: * indicates significance at the 5% level.

**Table 4:** Result of the Johansen cointegration test.

Lag	Number of Cointegrating Vectors	Trace Statistic	Maximum Eigenvalue Statistic	Coefficient of Cointegration	Whether the Residual of the VECM is Autocorrelated
4	0	149.8226	147.4186	-	-
	1	2.4040 *	2.4040 *	(1, −0.98808 *)	Yes
7	0	123.5055	121.1053	-	-
	1	2.4003 *	2.4003 *	(1, −0.98806 *)	No

Note: * indicates significance at the 5% level.

**Table 5:** Result of the Granger causality test.

Hypothesis	Spot Index Is Not the Granger Cause of Futures Price	Futures Price Is Not the Granger Cause of the Spot Index
chi2	3.2992	6.2492 *

Note: * indicates significance at the 5% level.

**Table 6:** The last ten rows of the market data.

Time	Index	Future	159919.SZ	159925.SZ	510300.SH	510310.SH	510360.SH
2022/4/13 14:15	4168.53	4173.2	4.167	2.008	4.166	1.940	1.454
2022/4/13 14:20	4170.57	4171.8	4.167	2.007	4.166	1.940	1.455
2022/4/13 14:25	4166.27	4169.6	4.165	2.007	4.163	1.940	1.453
2022/4/13 14:30	4167.44	4173.6	4.166	2.008	4.165	1.940	1.452
2022/4/13 14:35	4158.73	4164.6	4.158	2.003	4.157	1.935	1.450
2022/4/13 14:40	4160.89	4166.0	4.160	2.005	4.160	1.937	1.451
2022/4/13 14:45	4155.08	4160.6	4.154	2.004	4.152	1.933	1.449
2022/4/13 14:50	4147.75	4153.0	4.147	2.000	4.146	1.932	1.446
2022/4/13 14:55	4146.25	4151.0	4.145	1.998	4.144	1.930	1.446
2022/4/13 15:00	4139.74	4147.8	4.143	1.997	4.139	1.928	1.443

Note: The first two columns show the prices of the spot index and futures index. The last five columns show the net value of each ETF, respectively. Due to space limitations, here, we only show the last ten rows of the sample dataset.

**Table 7:** *TE* and *R*^2^ of each portfolio.

No.	Weight in Portfolio			*TE*	*R* ^2^
	159919.SZ	510300.SH	510310.SH		
1	91.45%			0.00061	0.8551
2		93.71%		0.00057	0.8720
3			90.46%	0.00067	0.8244
4	37.62%	57.35%		0.00054	0.8855
5	57.95%		36.54%	0.00057	0.8748
6		66.01%	29.62%	0.00054	0.8842
7	28.60%	46.08%	21.28%	0.00053	0.8910

**Table 8:** Explanation of symbols and parameter setting of Equation (3).

Symbol	Meaning	The Method to Decide the Parameter
*t*	Trading Day of Opening Positions	The futures contract we adopted is CSI 300 futures. In order to simplify the interval, we set T−t = 1/12.
*T*	Contract Expiration Date	
$S_{t}$	Spot Price on *t*th Day	The 5 min spot price of CSI 300.
$F_{t}$	Future Price on *t*th Day	The 5 min market price of CSI 300 futures.
$C_{s}$	Trading Cost and Impact Cost of Spot Trading	Trading cost was set as 0.025%, which is regulated by security companies. Impact cost was calculated by the net asset of each ETF, which compares the total scale between the ETFs and the whole market.
$C_{f}$	Trading Cost of Future Trading	As disclosed on the China Financial Futures Exchange (CFFE), the trading cost of index futures is 0.0023%.
$C_{\mathrm{fc}}$	Impact Cost of Future Trading	Set impact cost of futures trading as 0.5%, which is long-run statistical data.
*b*	Margin Ratio	As disclosed on the CFFE, the margin ratio of index futures is 8%.
D	Dividend	D=d*S_t_*(1 + *r*)^*T*−*t*^, d is the dividend rate.
r	Risk-Free Interest Rate	Using short-term treasury rate as risk-free interest rate is common. Therefore, we imported the Chinese 1-year treasury yield as the risk-free interest rate from Investing.com.
$r_{b}$	Rate of Borrowed Fund	Import overnight SHIBOR as rate of borrowed fund from Investing.com.
$r_{c}$	Rate of Security Loan	$r_{c}$ was set as 6.99% which is the lowest rate we could find in the security market.

**Table 9:** Different $RP_{n}$ and total trade return.

*RP_n_*	Total Return	Number of Trades
0.81480	−20.50%	1
0.90000	26.34%	54
0.91000	24.33%	55
0.92000	29.13%	60
0.93000	36.43%	64
0.94000	32.74%	66
0.95000	40.88%	69
0.96000	42.87%	88
0.97000	71.81%	138
0.98000	109.50%	307
0.99000	152.72%	587
0.99885	216.21%	938
0.99886	216.65%	939
0.99887	216.03%	941
1.00000	216.05%	983

**Table 10:** Parameter setting of machine learning model.

Algorithm	Parameter Setting
LSTM	num_lstm_layer = 2, learning_rate = 0.001, epoch = 500, optimizer = Adam, batch_size = 50
BPNN	learning_rate = 0.001, epoch = 500, optimizer = SGD, batch_size = 50
XGBoost	learning_rate = 0.01, max_depth = 5, n_estimator = 500
LASSO	Alpha = 0.00001

**Table 11:** Performance of each algorithms.

Algorithm	*RMSE*	*MAPE*	*R^2^*
LSTM	0.00813	0.70%	92.09%
BPNN	0.01314	1.00%	80.60%
XGBoost	0.01167	0.88%	86.69%
LASSO	0.01087	0.81%	88.34%

**Table 12:** Arbitrage return using machine learning models based on the test set.

Algorithm	Arbitrage Return	Number of Trades
Real Data	58.25%	307
LSTM	58.18%	277
BPNN	15.47%	161
XGBoost	−8.91%	50
LASSO	1.87%	56

**Table 13:** Performance of each algorithm under bull market.

Algorithm	*RMSE*	*MAPE*	*R^2^*
LSTM	0.01789	1.46%	70.70%
BPNN	0.02019	1.76%	62.67%
XGBoost	0.01498	1.28%	79.44%
LASSO	0.00443	0.37%	98.20%

**Table 14:** Performance of each algorithm under bear market.

Algorithm	*RMSE*	*MAPE*	*R^2^*
LSTM	0.00854	0.71%	94.21%
BPNN	0.01137	0.88%	89.72%
XGBoost	0.00910	0.60%	93.42%
LASSO	0.00410	0.34%	98.66%

## Data Availability

The data used to support the findings of this study are available from the corresponding author upon request.

## Appendix A

In our research, we chose one linear model, one ensemble learning model, one neural network model, and one deep learning model, which are all briefly introduced below.

### Appendix A.1. Least Absolute Shrinkage and Selection Operator

Being widely used in data dimension reduction and time series forecasting, LASSO is popular because of its simplicity and promising performance. The core idea of LASSO is adding a penalty term to the target of OLS so that regression shrinkage and selection can be solved [47]. The target of LASSO can be described as follows:

(A1) $$\sum_{i=1}^{n}{\left(y_{i}-\sum_{j}x_{\mathrm{ij}}\beta_{j}\right)}^{2}+\lambda \sum_{j=1}^{p}\left|\beta_{j}\right|$$

Here, *x_ij_* is the explanatory variable, *y_j_* is the explained variable, $\beta_{j}$ is the estimator, and λ is the learning rate.

### Appendix A.2. Extreme Gradient Boosting

Ensemble learning is one of the most-advanced machine learning models, where XGBoost, proposed by Chen and Guestrin in 2016 [48], is representative. The core principle of XGBoost is using the gradient boosting method to transform a weak classifier into a strong one. Due to its promising effectiveness and high efficiency, XGBoost has been widely used in time series forecasting [49], feature analysis [50], and so on.

Assume that ${\hat{y}}_{i}$ is the predicted value and *X_i_* is the input matrix. Then, ${\hat{y}}_{i}$ can be represented by Equation (A2):

(A2) $${\hat{y}}_{i}=\overset{N}{\underset{j=1}{\sum }}f_{j}(X_{i})\;,f_{j}\in F$$

where *F* represents a function space of the decision tree. The target is to minimize the loss function shown in Equation (A3):

(A3) $$L^{\left(t\right)}=\overset{n}{\underset{i=1}{\sum }}l\left({\hat{y}}_{i},y_{i}\right)+\Omega (f_{t})$$

where $\Omega \left(f\right)=\gamma t+\frac{1}{2}\lambda {\left||w|\right|}^{2}$ and *t* is the number of leaf nodes of a decision tree.

### Appendix A.3. BP Neural Network

The Back Propagation (BP) neural network, first proposed by Rumelhart and McClelland in 1986, is a gradient descent approach capable of optimizing the weight and threshold through error. However, the improved BPNN containing parameter optimization is more common in practice. For example, Wang et al. adopted GA-BP to forecast wind speed [51]. Du et al. also used GA-BP to assess the financial risk of Internet credit [52]. In recent years, however, the BPNN has been usually regarded as a benchmark model.

Three layers make up a typical BPNN: input, hidden, and output. Assume that the input and output data are *x* and *y*, respectively. The weight of neurons in the hidden layer and output layer is set as *w* and *v*. If *d* is the expected output, then the *Error* of the model can be described by the following:

(A4) $$\mathrm{Error}=\frac{1}{2}\overset{n}{\underset{k=1}{\sum }}{{(d}_{k}{-y}_{k})}^{2}$$

where *y_k_* is derived by Equation (A5) and *f* is the activation function.

(A5) $$y_{k}=f(\overset{n}{\underset{j=1}{\sum }}v_{\mathrm{jk}\;}f(\overset{m}{\underset{i=1}{\sum }}w_{\mathrm{ij}}{\;x}_{i}))$$

If the Error fails to hit the target, the neural network will adjust *w* and *v*. The adjustment amount of $\Delta w_{ij}\;$ and $\;\Delta v_{jk}$ is determined as follows:

(A6) $$\Delta w_{\mathrm{ij}}=-\eta \frac{\partial Error}{\partial w_{\mathrm{ij}}}\;i=1,2,\ldots ,m,\;j=1,2,\ldots ,n$$

(A7) $$\Delta v_{\mathrm{jk}}=-\eta \frac{\partial Error}{\partial v_{\mathrm{jk}}}\;k=1,2,\ldots ,n,\;j=1,2,\ldots ,n$$

where η is the learning rate. The iteration will come to an end once the neural network calculates for a predetermined number of cycles till the Error hits the target.

### Appendix A.4. Long Short-Term Memory Neural Network

In 1997, Sepp Hochreiter and Jürgen Schmidhuber [34] firstly proposed the long short-term memory neural network, which can judge the importance of previous information during the process of iteration so that the disappearance of the gradient or the phenomenon of gradient explosion caused by the long-term dependence of the RNN can be solved. Thereafter, LSTM has become a promising model to conduct time series forecasting. Various applications of LSTM can be witnessed in the past literature including traffic speed prediction [53], image classification [54], and so on. In the financial fields, LSTM and its improved model have shown an outstanding ability to forecast stock prices [55,56,57].

The mechanism of LSTM is as follows. Assume that $x_{t}$ is the input data, $c_{t}$ is a candidate for the updates, and $h_{t}$ is the output data of the LSTM neural network. The weight matrices and offset parameters used later are denoted as $M_{\mathrm{xf}}\;{,\;M}_{\mathrm{hf}}\;{,\;M}_{\mathrm{xi}}\;{,\;M}_{\mathrm{hi}}\;{,\;M}_{\mathrm{xc}}\;{,\;M}_{\mathrm{hc}\;}{,\;M}_{\mathrm{xo}}{,\;M}_{\mathrm{ho}}$, and $p_{f}\;{,\;p}_{i}\;{,\;p}_{c}\;{,\;p}_{o}$, respectively. The subscript here means different matrices and parameters used in different part. For example, *M_xf_* means the matrix of input data *x* in the forget gate, and *p_f_* means the offset parameter used in the forget gate.

Firstly, the forget gate is the basis of LSTM. A vector value is output through the transformation of the *sigmoid* function, and its value determines whether the information should be remembered. The closer it is to 1, the more information should be retained. Its mathematical expression is shown in Equation (A8).

(A8) $$f_{t}{=\mathrm{sigmoid}\;(\;M}_{\mathrm{xf}}\;x_{t}{+M}_{\mathrm{hf}}\;o_{t-1}{+p}_{f}\;)$$

Next, the input gate is constructed, which is usually composed of two parts. One selects information using the *sigmoid* function, while the other creates candidate state $\tilde{c_{t}}$ using the *tanh* function as follows:

(A9) $$i_{t}{=\mathrm{sigmoid}\;(\;M}_{\mathrm{xi}}{\;x}_{t}{+M}_{\mathrm{hi}}{\;o}_{t-1}{+p}_{i}\;)$$

(A10) $$\tilde{c_{t}}{=\tanh\;(\;M}_{\mathrm{xc}}{\;x}_{t}{+M}_{\mathrm{hc}}{\;o}_{t-1}{+p}_{c}\;)$$

The cell status is updated to obtain new information $c_{t}$, as shown in Equation (A11).

(A11) $$c_{t}{=f}_{t}{\;{}^{\circ}\;c}_{t-1}{+i}_{t}\;{}^{\circ}\;\tilde{c_{t}}$$

Finally, the output gate is built. The information gathered in the first two steps is contained in the output gate and is calculated as follows:

(A12) $$h_{t}{=\mathrm{sigmoid}\;(\;M}_{\mathrm{xo}}{\;x}_{t}{+M}_{\mathrm{ho}}{\;o}_{t-1}{+p}_{o}\;)$$

(A13) $$o_{t}{=h}_{t}{\;{}^{\circ}\;\tanh\;(c}_{t})$$
